# Immunoglobulin G subclass and antibody avidity responses in Malian children immunized with *Plasmodium falciparum* apical membrane antigen 1 vaccine candidate FMP2.1/AS02_A_

**DOI:** 10.1186/s12936-019-2637-x

**Published:** 2019-01-18

**Authors:** Andrea A. Berry, Eric R. Gottlieb, Bourema Kouriba, Issa Diarra, Mahamadou A. Thera, Sheetij Dutta, Drissa Coulibaly, Amed Ouattara, Amadou Niangaly, Abdoulaye K. Kone, Karim Traore, Youssouf Tolo, Vladimir Mishcherkin, Lorraine Soisson, Carter L. Diggs, William C. Blackwelder, Matthew B. Laurens, Marcelo B. Sztein, Ogobara K. Doumbo, Christopher V. Plowe, Kirsten E. Lyke

**Affiliations:** 1 Center for Vaccine Development and Global Health, University of Maryland School of Medicine, Baltimore, MD USA; 2 University of Sciences, Techniques, and Technologies, Bamako, Bamako, Mali; 3 Walter Reed Army Institute of Research, Silver Spring, MD USA; 4 United States Agency for International Development, Washington, DC USA; 5 Present Address: Warren Alpert Medical School, Brown University, Providence, RI USA; 6 Present Address: Duke Global Health Institute, Duke University, 310 Trent Drive, Durham, NC USA

**Keywords:** Malaria vaccine, Adjuvant, Natural exposure, Avidity, Immunoglobulin G subclass, Area under the curve

## Abstract

**Background:**

A malaria vaccine based on *Plasmodium falciparum* apical membrane antigen 1 (AMA1) elicited strain specific efficacy in Malian children that waned in the second season after vaccination despite sustained AMA1 antibody titers. With the goal of identifying a humoral correlate of vaccine-induced protection, pre- and post-vaccination sera from children vaccinated with the AMA1 vaccine and from a control group that received a rabies vaccine were tested for AMA1-specific immunoglobulin G (IgG) subclasses (IgG1, IgG2, IgG3, and IgG4) and for antibody avidity.

**Methods:**

Samples from a previously completed Phase 2 AMA1 vaccine trial in children residing in Mali, West Africa were used to determine AMA1-specific IgG subclass antibody titers and avidity by ELISA. Cox proportional hazards models were used to assess correlation between IgG subclass antibody titers and risk of time to first or only clinical malaria episode and risk of multiple episodes. Asexual *P. falciparum* parasite density measured for each child as area under the curve were used to assess correlation between IgG subclass antibody titers and parasite burden.

**Results:**

AMA1 vaccination did not elicit a change in antibody avidity; however, AMA1 vaccinees had a robust IgG subclass response that persisted over the malaria transmission season. AMA1-specific IgG subclass responses were not associated with decreased risk of subsequent clinical malaria. For the AMA1 vaccine group, IgG3 levels at study day 90 correlated with high parasite burden during days 90–240. In the control group, AMA1-specific IgG subclass rise and persistence over the malaria season was modest and correlated with age. In the control group, titers of several IgG subclasses at days 90 and 240 correlated with parasite burden over the first 90 study days, and IgG3 at day 240 correlated with parasite burden during days 90–240.

**Conclusions:**

Neither IgG subclass nor avidity was associated with the modest, strain-specific efficacy elicited by this blood stage malaria vaccine. Although a correlate of protection was not identified, correlations between subclass titers and age, and correlations between IgG subclass titers and parasite burden, defined by area under the curve parasitaemia levels, were observed, which expand knowledge about IgG subclass responses. IgG3, known to have the shortest half-life of the IgG subclasses, might be the most temporally relevant indicator of ongoing malaria exposure when examining antibody responses to AMA1.

**Electronic supplementary material:**

The online version of this article (10.1186/s12936-019-2637-x) contains supplementary material, which is available to authorized users.

## Background

A malaria vaccine is regarded as an essential tool towards the global eradication of malaria, and short of this ambitious goal, an effective blood stage malaria vaccine would prevent disease and death [1, 2]. Predicting and evaluating efficacy in malaria vaccine trials would be simplified with a reliable correlate of protection; however, identification of immune correlates of protection has proven elusive [3].

A previous clinical trial tested a blood stage malaria vaccine composed of a recombinant apical membrane antigen 1 (AMA1) formulated with the AS02_A_ adjuvant system (FMP2.1/AS02_A_) in Malian children. Children who received the vaccine had strain-specific protective efficacy and had increased AMA1 titers that were sustained for 24 months; however, no clinical efficacy was observed in the second season after vaccination, even though titers remained elevated [4, 5]. The short-lived efficacy of this vaccine cannot be explained by waning overall antibody titer, motivating exploration of other potential immune correlates of protection.

Total immunoglobulin G (IgG) is comprised of IgG subclasses named in order of relative abundance, IgG1, IgG2, IgG3, and IgG4 [6]. Differences in structural composition determine functionality: IgG1 and IgG3 are associated with T-cell dependent, cytophilic activity and typically recognize proteins [6]. They are the predominant subclasses observed in antibody dependent cellular cytotoxic responses to blood-stage merozoite antigens [7–14]. In contrast, IgG2 predominantly binds bacterial capsular polysaccharide antigens, which are typically T-cell independent. IgG4 binds allergens and chronically persistent antigens [15] and is, therefore, thought to exert anti-inflammatory effects.

Another humoral immune response characteristic that might influence protection is binding strength of antibodies to their targets, or avidity. This measure of intrinsic antibody function may distinguish effective from ineffective responses when total immunoglobulin quantities are equivalent. Avidity has been shown to differ across malaria transmission settings and to be associated with different clinical presentations [9, 16]. Low avidity has also been found to predict the failure of licensed vaccines for other infectious diseases [17], and although results vary across vaccine candidates, high avidity has been associated with vaccine-induced protection from malaria [18].

The hypothesis explored was that increased levels of cytophilic antibodies (IgG1 or IgG3) in malaria vaccine recipients and increased antibody avidity would be associated with protection from clinical episodes of malaria.

## Methods

### Study participants and trial design

The study was conducted in Bandiagara (population ~ 14,000), a rural town in Mali, West Africa that has intense seasonal transmission of *P. falciparum* malaria from July to November with a peak of up to 60 infective mosquito bites per person per month in August or September [19]. A randomized, double-blind, controlled Phase 2 trial of safety and efficacy against clinical malaria was conducted in 400 children from ages 1–6 years old in 2007–2008 and has been previously described [5]. Briefly, children were randomized in a 1:1 ratio to receive FMP2.1/AS02_A_, which comprises recombinant AMA1 based on the 3D7 *P. falciparum* strain formulated with GSKBio’s adjuvant system AS02_A_, or unadjuvanted rabies vaccine as a control, as three vaccinations given intramuscularly 1 month apart. Participants were followed for 2 years. The primary endpoint was a clinical episode of malaria, defined as fever (axillary temperature of ≥ 37.5 °C) with *P. falciparum* density of ≥ 2500 parasites/mm^3^ visualized on a thick blood smear. Efficacy was measured 6 months after final vaccination, or day 240. The primary analysis was of the first clinical malaria episode in an individual; a secondary analysis included multiple episodes in an individual. Exploratory efficacy endpoints included cumulative asexual *P. falciparum* parasite density measured for each child as the total area under the curve (AUC).

### Pilot study

Ten FMP2.1/AS02_A_ recipients and 10 controls who received rabies vaccine were randomly selected from all 400 participants for whom the previously measured day 90 total IgG titer was in the 25th–75th percentile for their respective groups (n = 98 and 96, respectively). Avidity and IgG subclass ELISAs were performed for samples from day 0, 90, and 150 for these subjects.

### Main study

90 FMP2.1/AS02_A_ recipients and 30 controls who had not already been included in the pilot study and from whom sera from appropriate time points were readily available (n = 177 from the malaria vaccine group, n = and 173 from the control group) were randomly chosen from the study population. Evaluable data was obtained from 85 vaccinees and 29 controls. Blinding to vaccine assignments was maintained until all assays were complete. IgG subclass ELISAs were performed on samples from days 0, 90, and 240.

### Positive control

A pool of sera from fifteen semi-immune adults obtained during peak malaria season who participated in a Phase 1 clinical trial of FMP1/AS02_A_, a merozoite surface protein-1 vaccine [20], was used as a positive control to monitor for batch effects and for construction of standard curves.

### ELISA methods

Ninety-six-well round-bottom Immulon 2 plates (Thermo Fisher Scientific, Waltham, MA) were coated with 50 µg of FMP2.1 diluted in phosphate buffered saline (PBS) to a total volume of 100 µL per well and incubated at 37 °C for 3 h. Plates were washed with PBS 0.05% Tween-20 (PBST) six times by a Biotek ELx405 automated plate-washer (Biotek, Winooski, VT), blocked with 10% nonfat milk by mass (Quality Biological, Gaithersburg, MD) in PBS, and incubated at 4 °C overnight. After washing, two-fold serum dilutions were tested in duplicate in PBS 0.05% Tween-20 with 10% nonfat milk (PBSMT). Plates were incubated for 2 h at 37 °C and then washed. For avidity assays, 100 µL of 5 M urea in PBST or 100 µL of PBST were added to wells and allowed to incubate at room temperature for 30 min. After washing, 100 µL of horse radish peroxidase (HRP)-conjugated goat anti-human IgG diluted with PBSMT to 1:10,000 (Jackson ImmunoResearch Laboratories, West Grove, PA) for avidity assays or subclass-specific HRP-conjugated sheep-anti-human secondary antibodies (The Binding Site, Birmingham, UK) diluted in PBSMT at concentrations of 1:400, 1:200, 1:250, and 1:100 for IgG1, IgG2, IgG3, and IgG4, respectively, were added to all wells. Plates were incubated at 37 °C for 1 h and then washed. For developing, 3,3′,5,5′-tetramethylbenzidine (KPL, Gaithersburg, MD) was allowed to equilibrate to room temperature and 100 µL was added to each well. Plates were placed in the dark for 15 min. The reaction was stopped with 100 µL per well of 1 M phosphoric acid, and optical densities (OD) were read by a Spectramax M2 plate-reader (Molecular Devices, Sunnyvale, CA) at 450 nm. Titers were calculated from linear regression curves created from positive control standards run on each plate, and defined as the reciprocal of the serum dilution that produced an OD of 0.2 above the blank. Avidity index was calculated as the ratio of the titer of the urea assay to the PBST assay. Cytophilic ratios were calculated as the sum of IgG1 and IgG3 titers divided by the sum of IgG2 and IgG4 titers.

### Statistical analysis

#### Comparison of titers across groups and time points

Log_10_-transformed IgG subclass titers, cytophilic ratios, avidity indices as well as differences between time points for these variables were compared using a two-tailed two-sample student’s t-test; a Kruskal–Wallis test was used for comparisons among age strata.

#### Correlation between IgG subclass and risk of malaria episode

For analysis of time to first or only clinical malaria episode, Cox proportional hazards models, using the Firth penalized likelihood to reduce bias [21], were fit with log_10_-transformed IgG subclass antibody titer as the explanatory variable, day of episode as the censoring variable, and survival to episode as the response variable. The models were fit with and without age as a covariate. The log-transformed titer was used to reduce the influence of extreme values. Subjects who had a clinical malaria episode or any parasitaemia (including illnesses for which anti-malarials were administered but that did not meet the fever and/or parasitaemia thresholds defined by the primary study outcome, as well as actively detected asymptomatic parasitaemia) before day 90 were excluded from the models, as the day 90 antibody responses would reflect both vaccination and infection. As a post hoc assessment of statistical power, for the AMA1 vaccine group, minimum detectable hazard ratios were calculated that took into account the number of clinical malaria illnesses (events) and standard deviations of each subclass IgG titer. The study had 80% power to detect a significant association between IgG subclass titer and hazard of clinical malaria episode when the hazard ratio varied from 1.61 to 4.03 (for risk factors), or from 0.62 to 0.25 (for protective factors), depending on the subclass titer and time point.

#### Correlation between IgG subclass and multiple episodes of malaria

For analysis of multiple episodes, a Cox proportional hazards gap time model with common coefficients for each episode, using the Firth penalized likelihood function, was used to assess the association of the log_10_-transformed day 90 IgG-subclass titer with clinical malaria [22].

#### Correlation between IgG subclass and level of parasitaemia

Asexual *P. falciparum* parasitaemia measured as AUC was estimated using the trapezoidal rule for each participant from day 90 to day 240 (AUC_d90–240_) and for control volunteers from time of enrollment to day 90 (AUC_d0–90_). As specified in the statistical analysis plan of the study protocol, all recorded episodes of parasitaemia were included regardless of symptom presence or absence, and parasite density was assumed to decline linearly to zero 3 days after a treated malaria episode [5]. IgG subclass titers were correlated with AUC_d90–240_ for the malaria vaccine group and the control group and AUC_d0–90_ for the control group, and Spearman’s rank correlations were calculated.

Statistical analysis was performed using SAS software, version 9.4 for Windows (SAS Institute, Cary, NC); GraphPad Prism version 6.0 for Windows (GraphPad Software, La Jolla, CA); and R version 3.4.4 with RStudio version 1.0.136 and packages tidyverse, ggplot2, ggbeeswarm, scales, and grid. Results with a P-value of less than 0.05 were considered statistically significant. No adjustment was made for multiple comparisons.

## Results

In children immunized on days 0, 30 and 60 with either the AMA1 vaccine or rabies vaccine as a control, avidity levels did not change significantly from day 0 to day 90 or from day 90 to day 150 and did not differ when comparing 10 AMA1 vaccinees to 10 children from the control group (Table 1). Based on the results of this pilot study, further avidity studies were not performed.

**Table 1:** Avidity index^a^ for pilot study of FMP2.1/AS02_A_ vaccinees and controls

	Day 0	Day 90	Day 150
FMP2.1/AS02_A_ (n = 10)			
Average (%)	35	31	30
Minimum (%)	9	15	15
Maximum (%)	89	56	52
Rabies (n = 10)			
Average (%)	21	26	23
Minimum (%)	9	5	3
Maximum (%)	47	52	48
*P* value^b^	0.26	0.19	0.078

^a^Avidity index is the ratio of the endpoint titer of the urea assay to the endpoint titer of the PBS-tween assay

^b^Mann–Whitney–Wilcoxon rank sum test *P* value

**Fig. 1:**
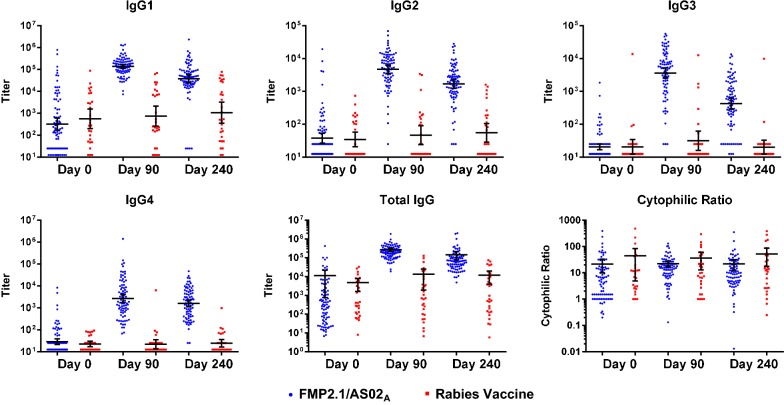
IgG subclass titers, total IgG titers, and cytophilic ratios. Depicted are geometric mean titers (GMT) and cytophilic ratios for 85 FMP2.1/AS02A vaccinees (blue) and 29 rabies vaccine controls (red) with 95% confidence intervals at days 0, 90, and 240. Vaccinations were administered on days 0, 30, and 60. Cytophilic ratios were calculated as the sum of the IgG1 and IgG3 titers divided by the sum of the IgG2 and IgG4 titers

As previously observed for whole IgG titers, in both the pilot study and the main study, baseline anti-AMA1 antibody titers for all four IgG subclasses in the FMP2.1/AS02_A_ group increased after vaccination and remained elevated 180 days after the last vaccination (Fig. 1, Additional file 1). In addition, at each time point after day 0, post-vaccination titers of all four IgG subclasses tested were significantly greater in the malaria vaccine group as compared with the control group. The total IgG titer for the subset of the FMP2.1/AS02_A_ and control groups presented here had similar magnitude and kinetics to the total IgG titers from all 400 participants in the original clinical trial [7].

**Fig. 2:**
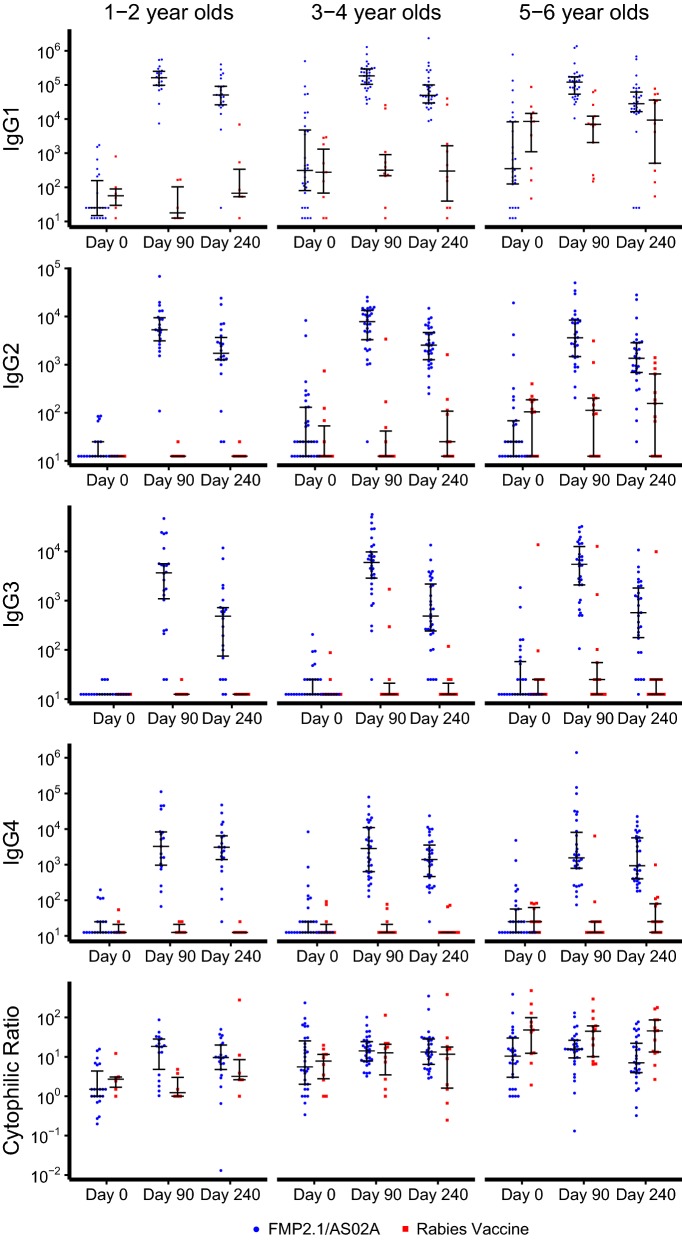
IgG subclass titers and cytophilic ratios across three age strata. IgG subclass titers for vaccine recipients (blue) and control subjects (red) with geometric mean titers and 95% confidence intervals at day 0, 90, and 240, grouped by age strata. Vaccinations were administered on days 0, 30, and 60. Cytophilic ratios were calculated as the sum of the IgG1 and IgG3 titers divided by the sum of the IgG2 and IgG4 titers

Subclass titers for each time point were compared across age strata (1–2 years, 3–4 years, and 5–6 years; Fig. 2, Additional file 2). At day 0, before vaccination, compared with the youngest age stratum, the older age strata of both the control and AMA1 vaccine groups had higher titers of IgG1, IgG2, and cytophilic ratio. In addition, for the AMA1 vaccine group, IgG3 titers at baseline were higher in the older age strata. At day 90, the control group continued to demonstrate greater IgG1, IgG2, and cytophilic ratios in the older age strata. In the AMA1 vaccine group, which had day 90 (30 days post-vaccination) titers that were 1–2 logs greater than those in the control group, day 90 antibody subclass titer did not differ among age strata. The lack of differentiation among age strata by day 90 subclass antibody titers also held true when comparing volunteers who did not have a clinical episode before day 90. At day 240, titers for IgG1 in the AMA1 vaccine group and IgG1, IgG2, and IgG4 in the control group differed significantly by age strata.

To explore the association between log-transformed IgG subclass titer and risk of infection, Cox proportional hazards models were constructed in which any subject who had a clinical episode before day 90 was excluded from the model (n = 66 AMA1 vaccinees and n = 22 control subjects included). In addition, to ensure that each day 90 subclass titer reflected AMA1 vaccination only and not exposure to natural infection including asymptomatic parasitaemia, a model that excluded all parasitaemias before day 90 was also explored in the AMA1 vaccine group (n = 49). For all models tested in the malaria vaccine and control groups, no log-transformed subclass titer predicted decreased time to first or only clinical malaria episode (Table 2). Moreover, post-vaccination subclass titers were not associated with the occurrence of multiple clinical malaria episodes in either the AMA1 vaccine or the control groups (Table 3).

**Table 2:** Cox regression survival analysis^a^ of time to clinical malaria episode^b^ between days 90 and 240

Predictor variable	AMA1 vaccine group				Control group	
	No primary outcome episodes before day 90 (n = 66)		No parasitaemia before day 90 (n = 49)		No primary outcome episodes before day 90 (n = 22)	
	Hazard ratio^c^	*P*	Hazard ratio	*P*	Hazard ratio	*P*
log_10_ (IgG1 day 0)	1.15 [0.85, 1.55]	0.36	1.38 [0.88, 2.16]	0.16	1.17 [0.64, 2.14]	0.60
log_10_ (IgG2 day 0)	1.07 [0.65, 1.77]	0.80	0.92 [0.30, 2.79]	0.88	0.99 [0.29, 3.34]	0.98
log_10_ (IgG3 day 0)	1.27 [0.59, 2.74]	0.54	1.45 [0.55, 3.84]	0.45	0.77 [0.24, 2.48]	0.66
log_10_ (IgG4 day 0)	1.36 [0.82, 2.27]	0.24	1.59 [0.47, 5.46]	0.46	5.70 [0.58, 56.3]	0.14
log_10_ (cytophilic ratio^d^ day 0)	1.42 [0.80, 2.54]	0.23	1.93 [0.92, 4.02]	0.081	1.30 [0.50, 3.39]	0.60
log_10_ (IgG1 day 90)	1.07 [0.42, 2.77]	0.88	0.785 [0.21, 2.89]	0.72	1.20 [0.66, 2.18]	0.55
log_10_ (IgG2 day 90)	0.85 [0.46, 1.59]	0.62	0.73 [0.28, 1.90]	0.52	1.35 [0.43, 4.27]	0.61
log_10_ (IgG3 day 90)	1.81 [0.93, 3.51]	0.079	1.62 [0.73, 3.56]	0.23	0.94 [0.36, 2.43]	0.90
log_10_ (IgG4 day 90)	1.05 [0.67, 1.65]	0.84	0.91 [0.48, 1.73]	0.77	3.58 [0.16, 79.9]	0.42
log_10_ (cytophilic ratio day 90)	0.83 [0.40, 1.75]	0.63	1.06 [0.29,3.81]	0.93	1.21 [0.48, 3.09]	0.68

^a^Using Firth’s penalized likelihood

^b^Clinical malaria episode using the primary outcome definition, i.e., an episode of malaria with axillary temperature of ≥ 37.5 °C and asexual *P. falciparum* density of ≥ 2500 parasites/mm^3^ of blood

^c^Hazard ratio and 95% confidence intervals for risk of clinical malaria episode after day 90

^d^Cytophilic ratio is the sum of IgG1 and IgG2 titers divided by the sum of the IgG3 and IgG4 titers

**Table 3:** Cox regression analysis^a^ of time to multiple clinical malaria episodes^b^ between days 90 and 240

	AMA1 vaccine group		Control group	
	No primary outcome episodes before day 90 (n = 66)		No primary outcome episodes before day 90 (n = 22)	
Predictor variable	Hazard ratio^c^	*P*	Hazard ratio	*P*
log_10_ (IgG1 day 90)	1.17 [0.47, 2.93]	0.74	1.14 [0.65, 2.00]	0.66
log_10_ (IgG2 day 90)	0.92 [0.5, 1.70]	0.79	1.27 [0.43, 3.72]	0.66
log_10_ (IgG3 day 90)	1.89 [0.99, 3.61]	0.053	0.93 [0.35, 2.43]	0.87
log_10_ (IgG4 day 90)	1.06 [0.69, 1.63]	0.79	8.45 [0.61, 116.86]	0.11
log_10_ (cytophilic ratio^d^ day 90)	0.83 [0.40, 1.70]	0.61	1.05 [0.44, 2.53]	0.91

^a^Gap time models with common regression coefficients using Firth’s penalized likelihood

^b^Clinical malaria episode defined using the primary outcome definition, i.e., an episode of malaria with axillary temperature of ≥ 37.5 °C and asexual *P. falciparum* density of ≥ 2500 parasites/mm^3^ of blood

^c^Hazard ratio and 95% confidence intervals for risk of multiple clinical malaria episodes after day 90

^d^Cytophilic ratio is the sum of IgG1 and IgG2 titers divided by the sum of the IgG3 and IgG4 titers

Asexual *Plasmodium falciparum* parasite densities measured as area under the curve (AUC) were calculated to study correlations between parasite burden and IgG subclass levels. In the malaria vaccine group, not including those with clinical malaria episodes between days 0 and 90 (n = 66), the AUC_d90–240_ correlated positively with IgG3 at day 90 and IgG3 at day 240. In contrast, AUC_d90–240_ did not have a statistically significant correlation with any IgG subclass at any time point in the control arm when those with clinical malaria episodes between days 0 and 90 were excluded (n = 22) (Table 4). However, when the control arm was evaluated without censoring participants for having infections between days 0 and 90 (n = 29), AUC_d0–90_ correlated positively with IgG1, IgG2, IgG3, and IgG4 at day 90 and IgG2 and IgG3 at day 240; additionally AUC_d90–240_ correlated with IgG3 at day 240 (Table 5). Spearman’s rank correlations for IgG subclass titers for each time point and vaccination group were calculated—strong correlations were seen when comparing subclasses to each other except when comparing IgG3 to the other subclasses for the rabies group at day 0 and day 240 (Additional file 3).

**Table 4:** Correlation between area under the curve parasitaemia during study days 90–240 and IgG subclass titers

	FMP2.1/AS02_A_ vaccine group (n = 66)		Control group (n = 22)	
	R	P-value	R	P-value
IgG1 day 90	0.077	0.54	0.22	0.32
IgG2 day 90	0.026	0.84	0.11	0.64
IgG3 day 90	*0.29*	*0.019*	0.14	0.53
IgG4 day 90	− 0.057	0.66	0.22	0.33
IgG1 day 240	0.24	0.052	0.041	0.86
IgG2 day 240	0.13	0.28	0.11	0.62
IgG3 day 240	*0.32*	*0.010*	0.23	0.30
IgG4 day 240	− 0.16	0.20	0.24	0.28

Italic values denote significant results. Volunteers who were treated for a clinical malaria episode before day 90 were excluded from this analysis. R, Spearman correlation coefficient

**Table 5:** Correlation between area under the curve parasitaemia and IgG subclass titers in volunteers who received control vaccine

	Control group (n = 29)	
	R	P-value
AUC days 0–90		
IgG1 day 90	*0.48*	*0.009*
IgG2 day 90	*0.60*	*0.0005*
IgG3 day 90	*0.71*	< *0.0001*
IgG4 day 90	*0.57*	*0.001*
IgG1 day 240	0.25	0.19
IgG2 day 240	*0.39*	*0.036*
IgG3 day 240	*0.45*	*0.015*
IgG4 day 240	0.35	0.059
AUC days 90–240		
IgG1 day 90	0.28	0.14
IgG2 day 90	0.16	0.41
IgG3 day 90	0.28	0.15
IgG4 day 90	0.27	0.16
IgG1 day 240	0.14	0.47
IgG2 day 240	0.35	0.062
IgG3 day 240	*0.42*	*0.022*
IgG4 day 240	0.26	0.18

Italic values denote significant results

*R* Spearman correlation coefficient, *AUC* area under the curve

## Discussion

In this analysis of IgG subclass antibodies elicited in response to an AMA1 vaccine, the vaccine elicited robust, ≥ 2-log_10_ increases in all four IgG subclasses that peaked at day 90 (30 days post-vaccination) and then waned by day 240, but remained ≥ 1 log_10_ higher than pre-vaccination antibody responses. In contrast, over the malaria transmission season, the rabies vaccine control children had a steady increase in IgG subclass antibodies that remained 1–2 log_10_ lower than the antibody responses in the AMA1 vaccine group, representing the naturally occurring immune response to a blood stage malaria antigen over a malaria transmission season. In the control group, IgG subclass titers correlated with increasing age, reflecting cumulative acquisition of immunity. In the AMA1 vaccine group, these effects were not observed, presumably because the adjuvanted vaccine increased titers to all subclasses globally, such that the youngest children’s titers approached the levels of the oldest children. Indeed, children who received FMP2.1/AS02_A_ had a several-fold lower baseline than adults residing in the same area, but after vaccination they had total IgG titers that were similar in magnitude to those of adults who received the same vaccine [5, 23]—thus the fold-rise in IgG was much larger in children than in semi-immune adults, who have a greater degree of acquired humoral immunity from previous *P. falciparum* exposure.

The original hypothesis of this study was that increased levels of cytophilic antibodies (IgG1 or IgG3) in malaria vaccine recipients would be associated with protection from clinical episodes of malaria. However, increased IgG subclass titers were not associated with decreased risk of clinical episodes among children who received the AMA1 vaccine. By examining antibody responses in the control group, the effect of natural malaria exposure on subclass IgG titers was explored; however, no associations between day 90 (peak season) IgG subclass titers and risk of subsequent clinical malaria illness were identified.

The relationship between exposure to *P. falciparum* and subclass immunoglobulin titer was assessed by estimating the parasite burden for each volunteer, expressed as AUC parasitaemia. In the AMA1 vaccine group, for children who did not have clinical malaria episodes within the first 90 days (n = 66), AUC_d90–240_ correlated with increased IgG3 at the day 90 and day 240 time points, suggesting that IgG3 levels in the AMA1 vaccine group correlate with exposure to parasites. One explanation is that increased IgG3 levels at day 90 indicate ongoing malaria exposure and counterintuitively predict infection in the ensuing 150 days. This correlation was not seen in the control group; the smaller sample size of the control arm might have limited the power to detect this association.

When the association between IgG subclass titer and AUC parasitaemia in the control arm without censoring subjects who had clinical infections in the first 90 study days was examined, correlations were identified between IgG subclass titers and parasite burden attributable to natural exposure. These correlations were observable because of the larger sample size (n = 29 vs. n = 22) and examination of AUC_d0–90_ (rather than AUC_d90–240_), an exposure interval not explored in the AMA1 vaccine group because vaccination occurred within the 0–90 day interval. In a pre-planned exploratory analysis of the original vaccine trial, median AUC was lower in vaccines than controls, suggesting an overall reduction in parasite burden that did not translate into a statistically significant reduction in the hazard of clinical malaria episodes [5]. In the current study, the significant correlations between the control arm IgG subclass titers and AUC_d0–90_ support further evaluation the utility of AUC as a clinically relevant readout of malaria exposure. The finding that IgG3 at day 90 correlated with AUC_d90–240_ in both the AMA1 vaccine and control arms suggests that IgG3, which has the shortest half-life of the IgG subclasses, might be the most temporally relevant indicator of ongoing exposure. Thus IgG3 may be worthy of exploring as a biomarker for surveillance to detect populations experiencing ongoing or very recent exposure to help target malaria elimination interventions.

In other infectious diseases, humoral immune responses have served as correlates of protection [24, 25]. In malaria, antibodies have a proven functional role, although no generalizable and consistent correlates of vaccine-induced or naturally acquired protection have been established [26]. Naturally occurring human antibodies against AMA1 are associated with clinical protection from malaria [27] and have functional invasion inhibition activity [28], justifying a focus on humoral immunity when searching for AMA1 vaccine-induced immune correlates of protection [29]. Antibody avidity to full length VAR2CSA has been associated with absence of placental malaria in both high and low transmission settings in Cameroon [30, 31], and a study of adjuvanted circumsporozoite vaccine (RTS, S/AS01) showed increased avidity and protection when the third dose was fractional rather than full-dose [18]. But, the association between avidity and protection is not universal [32, 33], and some studies have observed a lack of correlation between avidity assays and other functional antibody assays [34, 35].

Overall, the results from this study demonstrate the robust humoral response elicited by the FMP2.1/AS02_A_ vaccine across all subclasses, and are among a handful of descriptions of antibody subclass and avidity responses to a candidate malaria vaccine. Although a correlate of protection based on antibody subclass or avidity was not observed for this vaccine, antibody subclass levels or avidity could be associated with protection for other malaria vaccines that exert broader efficacy. The lack of correlation of avidity or antibody subclass titer with protection despite the FMP2.1/AS02_A_ vaccine eliciting strain-specific efficacy, and the lack of correlation of a growth inhibition assay with protection [36] suggest that additional signatures of humoral immune responses could be explored. These could include post-vaccination antibody response epitope mapping (Bailey et al. pers. comm.), additional studies of antibody function [37], and transcriptomic investigations that explore somatic hypermutation and affinity maturation [38, 39].

## Conclusions

IgG subclass titers were not associated with vaccine-induced protection from subsequent malaria illness. However, older age correlated with increased IgG subclass titers in the control arm and not in the AMA1 vaccine arm, which reflects IgG subclass response to natural infection in the former and the adjuvant effect in the latter. IgG subclass titers were a marker of exposure that correlated with AUC parasitaemia levels. An association between IgG3 and subsequent parasite burden suggests that of the IgG subclasses, IgG3 may be the most temporally relevant indicator of ongoing malaria exposure when assessing AMA1 responses, and worthy of further exploration as a biomarker for surveillance in support of elimination efforts.

## Additional files

**Additional file 1.** AMA1 IgG subclass titers, total IgG titers, and cytophilic ratios for AMA1 vaccine and control groups.

**Additional file 2.** Effect of age strata on IgG subclass titer.

**Additional file 3.** Spearman’s Rank correlations of AMA1 IgG subclass titers grouped by time point and vaccine group.

## Abbreviations

AMA1: apical membrane antigen 1
AUC: area under the curve
GMT: geometric mean titer
HRP: horseradish peroxidase
IgG: immunoglobulin G
IRB: Institutional Review Board
NIAID: National Institute of Allergy and Infectious Diseases
NIH: National Institutes of Health (NIH)
PBS: phosphate buffered saline
OD: optical density
PBST: phosphate buffered saline and 0.05% Tween-20
PBSMT: phosphate buffered saline, 0.05% Tween-20, and 10% nonfat milk
